# The Silent Shunt: Traumatic Splenic AV Fistula Revealed and Treated Endovascularly

**DOI:** 10.15388/Amed.2025.32.2.10

**Published:** 2025-12-30

**Authors:** Gulshan Sharma, Resham Singh, Puneet Garg

**Affiliations:** 1 Department of Radiodiagnosis and Interventional Radiology, Vardhman Mahavir Medical College and Safdarjung Hospital; 2 Department of Radiodiagnosis and Interventional Radiology, Vardhman Mahavir Medical College and Safdarjung Hospital; 3 Department of Radiodiagnosis and Interventional Radiology, Vardhman Mahavir Medical College and Safdarjung Hospital

**Keywords:** arteriovenous fistula, spleen, trauma, endovascular, arterioveninė fistulė, blužnis, trauma, endovaskulinė embolizacija

## Abstract

**Background:**

Splenic arteriovenous fistula (AVF) is a rare vascular anomaly that can arise from trauma, aneurysmal rupture, or congenital conditions. Prompt diagnosis and management are crucial to preventing complications such as portal hypertension and high-output cardiac failure.

**Case Presentation:**

We present the case of a 62-year-old male with a traumatic splenic AVF resulting from a road traffic accident (RTA), associated with a Grade IV splenic injury and multiple pseudoaneurysms. The patient underwent successful endovascular coil embolization and experienced an asymptomatic follow-up.

**Conclusion:**

Endovascular coil embolisation is a minimally invasive and effective first-line treatment for traumatic splenic AVFs, particularly in hemodynamically stable patients, providing excellent outcomes and organ preservation.

## Abstract (it)

**Įvadas:**

Blužnies arterioveninė fistulė (AVF) yra reta kraujagyslių anomalija, ji gali atsirasti dėl traumos, aneurizmos plyšimo ar įgimtų sutrikimų. Greita diagnozė ir gydymas yra labai svarbūs siekiant išvengti komplikacijų, tokių kaip antai portinė hipertenzija ir didelis širdies nepakankamumas.

**Atvejis:**

Pristatome 62 metų vyro, patyrusio trauminę blužnies AVF dėl eismo įvykio (RTA), susijusio su IV laipsnio blužnies sužalojimu ir daugybe pseudoaneurizmų, atvejį. Pacientui buvo sėkmingai atlikta endovaskulinė embolizacija spirale ir po operacijos jis nesiskundė jokiais simptomais.

**Išvada:**

Endovaskulinė embolizacija spirale yra minimaliai invazinis ir veiksmingas pirminis trauminių blužnies AVF gydymo būdas, ypač stabilios hemodinamikos pacientams, užtikrinantis puikius rezultatus ir galimybę išsaugoti organus.

## Introduction

Splenic arteriovenous fistula (AVF) is an uncommon yet potentially life-threatening condition involving direct communication between the splenic artery and vein [1]. While congenital causes are often associated with connective tissue disorders such as Ehlers-Danlos and Rendu-Osler-Weber syndromes, acquired AVFs are more frequently linked to trauma [2], surgical intervention, or spontaneous rupture of splenic artery aneurysms, which are commonly observed in multiparous women and those of reproductive age [3]. Early identification through imaging and minimally invasive endovascular intervention is essential for optimal patient outcomes. Herein, we report a case of traumatic splenic AVF that was successfully managed with minimally invasive endovascular coil embolisation of the splenic artery.

## Case Presentation

A 62-year-old male was brought to the Emergency Department after a road traffic accident, presenting with left-sided chest and abdominal pain. A focused assessment with sonography in trauma (FAST) revealed moderate free fluid with internal echoes in the abdomen, suggesting hemoperitoneum.

**Figure 1:**
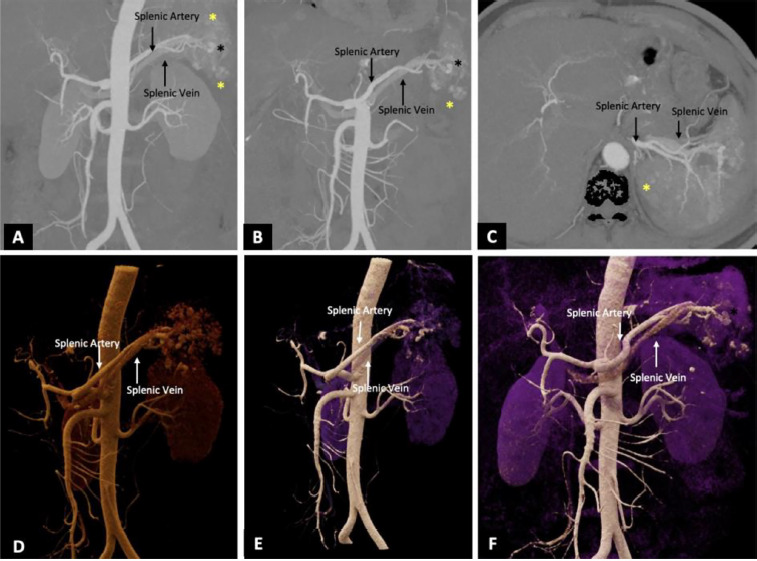
Maximum intensity projection (MIP) images in coronal (A, B) and axial (C) orientations, along with cinematic volume-rendered tomographic (VRT) images (D–F), reveal multiple contusions (the yellow asterisk) involving the lower pole of the spleen. In addition, there are pseudoaneurysms (the black asterisk) arising from the lower pole branch of the splenic artery, with early opacification of the splenic vein, suggestive of a splenic arteriovenous fistula

CT angiography (CTA) of the abdomen revealed multiple splenic contusions with over 30% non-enhancing parenchyma in the mid and lower poles, indicating devascularization. Several pseudoaneurysms were identified, along with early opacification of the splenic vein in the arterial phase – s/o splenic AVF (Figure 1, A–F).

The patient was hemodynamically stable. His BP was 133/82 mm Hg, and Spo2 was 94%; however, he had low haemoglobin (6 g/dl). Two units of packed red blood cells were transfused, and endovascular embolisation of the splenic artery was planned following a multidisciplinary discussion.

## Procedure

Under aseptic conditions, proper transfemoral arterial access was achieved by using a 6F vascular sheath. The celiac trunk and selective splenic artery angiogram revealed an abnormal intrasplenic contrast blush with multiple pseudoaneurysms from the lower pole branches of the splenic artery, demonstrating early opacification of the splenic vein. Super-selective catheterisation of the affected lower pole branch was performed by using a microcatheter. A frame coil (8 mm) was initially deployed to reduce the arterial flow, followed by several smaller coils measuring 2 mm and 3 mm in diameter to attain complete occlusion. Post-embolisation angiography showed no opacification of the lower pole branches, AVF, and pseudoaneurysms, while preserving the perfusion of the unaffected upper splenic pole.

**Figure 2:**
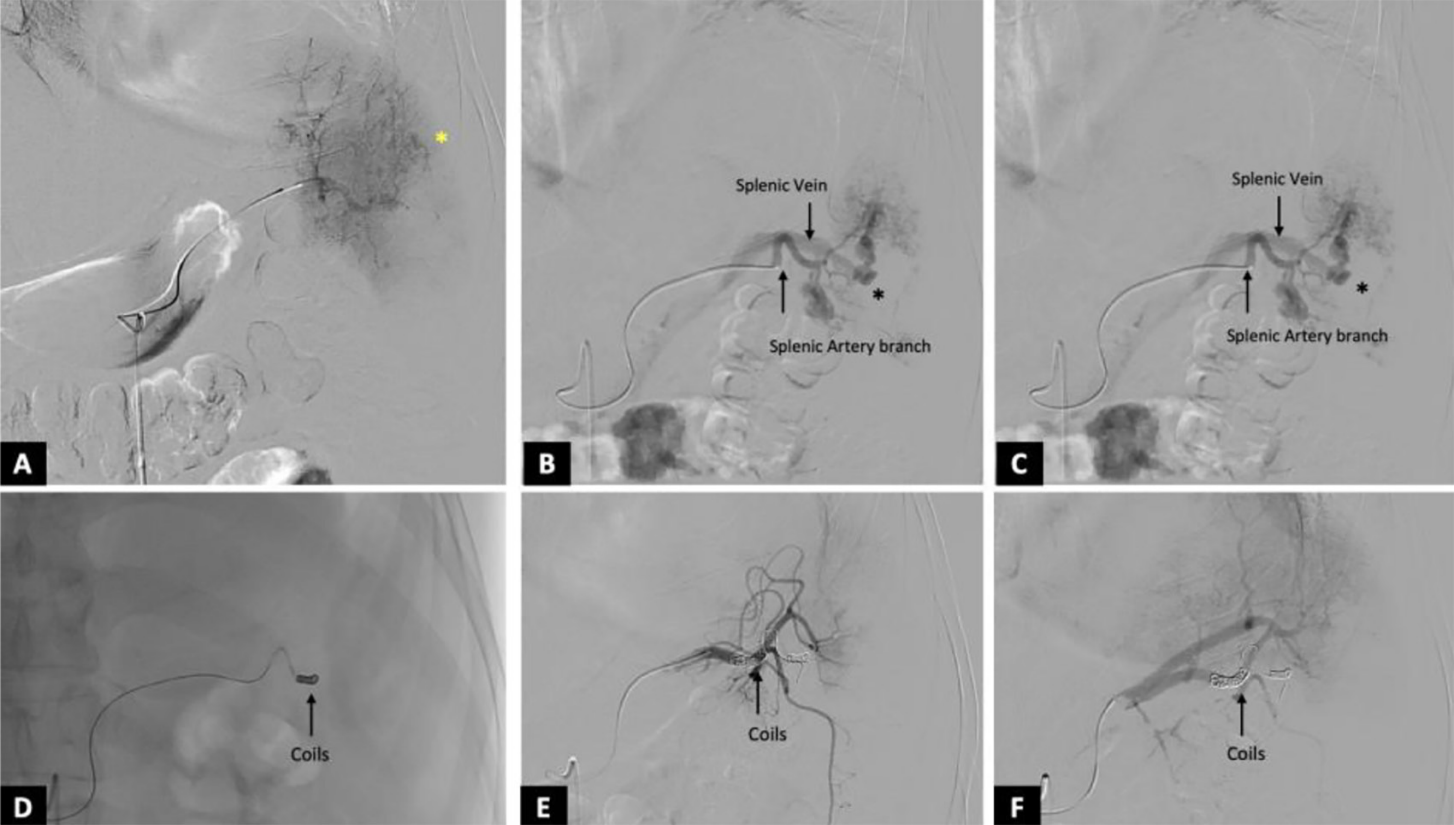
Figure 2A shows a selective run of the lower polar branch of the splenic artery showing multiple parenchymal blushes (the yellow asterisk). In addition, pseudoaneurysms (the black asterisk) arising from the lower pole branches with early opacification of the splenic vein are also observed, suggesting traumatic splenic artery pseudoaneurysms with an arteriovenous fistula. Successful coil embolisation of the lower pole branch was performed with a framing coil of 8 mm, followed by packing the frame coil with multiple 2mm and 3mm coils. The post-embolisation run revealed successful embolisation of the lower pole branch of the splenic artery with no opacification of the pseudoaneurysm. No early opacification of the splenic vein was seen, suggesting successful exclusion of the fistulous communication

## Outcome and Follow-up

The patient remained hemodynamically stable throughout the procedure and was discharged in good condition after four days. No splenectomy was needed, and follow-up sonography revealed no recurrence of the AVF or complications.

## Discussion

Traumatic splenic AVFs are rare but represent serious vascular injuries [1]. If left untreated, they may lead to rapid-onset portal hypertension, gastrointestinal bleeding, ascites, and high-output cardiac failure [1]. They can also be diagnosed via ultrasound and should be suspected in portal hypertensive patients without a history of liver cirrhosis. They appear as an anechoic, pulsatile mass near the splenic hilum, with dilatation of the splenic vein, and on colour Doppler study, as a lesion with reversed colour flow patterns, the so-called characteristic ‘yin-yang’ (a red and blue flow) pattern. Spectral Doppler interrogation shows high-velocity, low-impedance arterial and moderately pulsatile venous flow [4]. Imaging with CT angiography (CTA) is key to diagnosis and planning the intervention. On CTA, splenic AVF reveals early opacification of the splenic vein and associated pseudoaneurysms.

Most literature comparing endovascular repair and surgical approaches has focused on splenic artery pseudoaneurysms. While surgical options such as splenectomy or vascular ligation have historically been used, endovascular techniques are now the preferred first-line therapy [5]. Vascular ligation is difficult for AVFs due to their distal location, the formation of adhesions, and perisplenic collaterals [6]. Endovascular management offers advantages such as a minimally invasive nature, reduced morbidity, shorter hospital stays, and organ preservation.

Partial splenic artery embolisation has been advocated as a minimally invasive treatment alternative to surgery for splenic AVF [6]. The embolic agents of choice in splenic AVF are coils or a vascular plug. Coils are preferred over plugs in partial splenic embolisation as they offer easier distal deployment. In contrast, plugs are preferred for proximal splenic artery embolisation, such as in the setting of hypersplenism [7, 8].

The existing literature recommends using coils in AVF as they prevent non-target embolisation and minimise the washout of glue/particulate embolising agents [6, 7, 8]. In one case, both coils and n-butyl cyanoacrylate glue were employed as embolising agents in a splenic AVF, based on the reasoning that the coil decreases arterial flow, allowing the glue to settle and facilitate instantaneous closure of the AVF. However, splenic infarction was observed in follow-up imaging, although the patient remained asymptomatic and did not require further intervention [9].

The decision to use a specific embolic agent depends on the ability to access the target and the nature of the lesion. When accessible, arteriovenous fistulas and pseudoaneurysms are treated with coils and/or gelatin sponges to enhance hemostasis [10].

In our case, successful partial splenic embolisation was achieved while preserving the splenic functions of the patient, and no additional vaccination for pneumococcal or meningococcal infections was needed.

The occurrence of complications after endovascular treatment is uncommon. Possible complications include postembolisation syndrome, transient elevation of pancreatic enzymes, splenic infarction, infection, abscess, and, rarely, rupture of a pseudoaneurysm [10].

## Conclusion

Splenic AVF, although rare, should be considered in cases of high-grade splenic trauma. Early diagnosis and prompt intervention are essential. Endovascular coil embolisation offers a safe, effective, and minimally invasive treatment, reducing the need for splenectomy and its associated complications.
